# Plasma Protein Corona Fingerprinting Across Intraductal Papillary Mucinous Neoplasms and Pancreatic Ductal Adenocarcinoma: A Comparative Proof-of-Concept Study

**DOI:** 10.3390/cancers18203275

**Published:** 2026-10-11

**Authors:** Sara Elsa Aita, Vincenzo La Vaccara, Roberto Cammarata, Marco Talarico, Federica Giordano, Roberto Coppola, Damiano Caputo

**Affiliations:** 1 Operative Research Unit of Laparoscopic and Minimally Invasive Surgery, Fondazione Policlinico Universitario Campus Bio-Medico, Via Alvaro del Portillo, 200, 00128 Rome, Italy; 2 Operative Research Unit of General Surgery, Fondazione Policlinico Universitario Campus Bio-Medico, Via Alvaro del Portillo, 200, 00128 Rome, Italy; 3 Research Unit of General Surgery, Università Campus Bio-Medico di Roma, 00128 Rome, Italy

**Keywords:** pancreatic ductal adenocarcinoma, intraductal papillary mucinous neoplasm, protein corona, graphene oxide, plasma proteomic fingerprint, disease stratification, minimally invasive diagnostics

## Abstract

**Background/Objectives**: Surveillance of intraductal papillary mucinous neoplasms (IPMNs) provides a clinically relevant window in which biological changes associated with pancreatic ductal adenocarcinoma (PDAC) development may potentially be investigated before advanced disease occurs. Nanoparticle-enabled protein corona (PC) fingerprinting has shown analytical potential in PDAC, but the extent to which different nanoplatforms recover distinct information across the biologically proximate IPMN-PDAC spectrum remains unclear. This exploratory study compared three nanoplatform configurations to identify a candidate workflow for subsequent validation. **Methods**: Plasma from 22 patients with de novo PDAC (*n* = 11), IPMN (*n* = 6), or IPMN-derived PDAC (*n* = 5) was incubated with graphene oxide (GO) nanosheets, gold nanoparticles (AuNPs), or cationic DOTAP-DOPE liposomes. Wash-resistant PCs were analyzed by SDS-PAGE, quantified across predefined molecular weight (MW) regions, and evaluated using exploratory linear discriminant analysis with repeated stratified cross-validation. **Results**: The three nanoplatforms generated non-equivalent electrophoretic PC architectures. In this small exploratory cohort, GO showed the broadest pattern of nominal clinical-group-associated variation in relative MW-region contributions and yielded internal cross-validation AUC estimates of 0.955 for IPMN versus de novo PDAC, 0.767 for IPMN versus IPMN-derived PDAC, and 0.927 for IPMN-derived versus de novo PDAC. Corresponding AUCs were 0.606–0.700 for AuNPs and 0.621–0.691 for liposomes. **Conclusions**: GO provided the broadest group-associated variation and numerically highest internal discrimination estimates, supporting its prioritization for independent validation and prospective longitudinal evaluation in IPMN surveillance.

## Simple Summary

Intraductal papillary mucinous neoplasms (IPMNs) are pancreatic cystic lesions that can be detected and followed over time, offering an opportunity to monitor patients before advanced pancreatic cancer develops. Current surveillance relies mainly on imaging and clinical features, while blood-based markers could provide complementary biological information. In this exploratory study, plasma from patients with non-invasive IPMN, IPMN-derived pancreatic ductal adenocarcinoma (PDAC), and de novo PDAC was analyzed using three nanoparticle platforms. Each platform generated a different protein corona (PC) fingerprint, indicating that the analytical information recovered from plasma depends on the nano–bio interface. Graphene oxide (GO) showed the broadest differences among the clinical groups and was therefore prioritized for further study. These findings provide a basis for prospective and longitudinal evaluation of GO-based PC profiling as a potential complementary tool in IPMN surveillance.

## 1. Introduction

Pancreatic ductal adenocarcinoma (PDAC) accounts for the large majority of pancreatic malignancies and remains one of the most lethal solid tumors. Despite advances in treatment, long-term survival remains poor, largely because the disease is frequently diagnosed when curative resection is no longer feasible [1,2]. Early-stage PDAC is often clinically silent, and no blood-based test is currently validated for routine population screening. Conversely, outcomes improve substantially when tumors are detected at a small, organ-confined, and potentially resectable stage, emphasizing the need for strategies that can support earlier recognition of clinically relevant disease [3].

Intraductal papillary mucinous neoplasms (IPMNs) provide a particularly relevant clinical setting in this context. Unlike microscopic pancreatic intraepithelial neoplasia, IPMNs are detectable by imaging and can be monitored longitudinally before advanced invasive disease develops. Current management relies primarily on morphological and clinical criteria. The 2024 International Evidence-Based Kyoto Guidelines incorporate cyst morphology, main-duct caliber, mural nodules, symptoms, cytology, and other worrisome features or high-risk stigmata to guide surveillance and surgical management [4]. However, IPMNs have heterogeneous malignant potential: many lesions remain indolent, whereas a subset progresses toward advanced neoplasia [5,6]. The central clinical challenge is therefore to distinguish patients who can be safely monitored from those showing biologically relevant progression.

The oncological risk in patients with IPMN is not limited to malignant transformation of the monitored lesion. Pancreatic cancer may arise either through progression to IPMN-derived carcinoma or through development of a separate concomitant PDAC elsewhere in the gland. Surveillance must therefore consider not only changes in the index cyst but also the risk associated with the entire IPMN-bearing pancreas. In a large prospective cohort, long-term surveillance of patients with IPMN was associated with earlier detection of concomitant PDAC, higher rates of R0 resection, and improved survival compared with matched non-IPMN-associated PDAC cases [7]. These observations further support IPMN surveillance as a clinically meaningful window for earlier recognition of pancreatic malignancy.

IPMN-derived and conventional PDAC may also differ biologically. Molecular and immune differences have been reported between these tumor types [8], together with differences in treatment response and clinical outcome [9]. A complementary blood-based biomarker should therefore ideally provide biological information beyond structural changes in the monitored cyst and capture broader systemic alterations associated with pancreatic malignancy. Such an approach would not replace magnetic resonance imaging, endoscopic ultrasound, cytology, histopathology, or guideline-defined clinical criteria, but could add a molecular dimension to longitudinal risk assessment.

Available circulating biomarkers only partially address this need. Carbohydrate antigen 19-9 (CA 19-9) is widely used in pancreatic cancer management, but its diagnostic performance is limited by incomplete sensitivity and specificity and by factors including biliary obstruction, inflammation, tumor burden, and Lewis antigen status [10,11]. Circulating tumor DNA is detected less consistently in localized than in advanced disease [12], whereas combinations of genomic and protein biomarkers have demonstrated the value of integrating multiple circulating signals [13,14]. Multiplex protein signatures [15], lipidomic, metabolomic, and integrated multi-omics approaches [16,17,18] further support the broader concept that coordinated molecular patterns may provide information that cannot be captured by a single tumor-derived analyte.

Nanoparticle-enabled protein corona (PC) profiling represents one such pattern-based strategy. When nanoparticles (NPs) are exposed to plasma, proteins and other biomolecules adsorb to their surfaces and form a biomolecular corona, whose protein component can be experimentally recovered and analyzed [19,20,21]. Importantly, the resulting corona does not simply reproduce bulk plasma composition. NP physicochemical properties influence competitive biomolecular adsorption, causing different nanomaterials to selectively enrich distinct subsets of the circulating proteome [22,23,24]. NPs can therefore act as nanoscale fractionation interfaces, converting the same biological specimen into material-dependent molecular fingerprints that may reveal coordinated changes otherwise less evident in unfractionated plasma.

The analytical feasibility and discriminatory potential of PC-based blood testing have already been investigated in pancreatic cancer using lipid-based NPs, gold NPs (AuNPs), and graphene oxide (GO) [25,26,27,28]. Subsequent studies have explored multiplexed approaches, integration with conventional circulating markers, and standardized electrophoretic workflows [29,30,31,32]. However, most of this evidence derives from conventional PDAC-versus-control comparisons. Whether different nanoplatforms recover equivalent or distinct analytical information across non-invasive IPMN, IPMN-derived PDAC, and de novo PDAC remains insufficiently explored.

This represents a more stringent analytical setting than conventional cancer-versus-control classification. The three conditions arise within the same organ context and may share inflammatory, stromal, metabolic, and systemic alterations. Moreover, differences between groups may reflect not only invasive disease but also IPMN morphology, duct involvement, obstruction, inflammation, tumor burden, and other clinical characteristics. Their comparison therefore provides an appropriate exploratory setting in which to assess both platform dependence and clinical-group-associated structure in nanoparticle-enabled plasma fingerprints.

In the present paired, within-specimen exploratory study, plasma samples from patients with IPMN, IPMN-derived PDAC, and de novo PDAC were independently interrogated using GO nanosheets, AuNPs, and cationic DOTAP–DOPE liposomes. The primary objective was to determine whether these three analytical configurations generated equivalent or non-equivalent electrophoretic PC fingerprints from the same plasma specimens and to identify a candidate workflow for subsequent validation. As a secondary exploratory objective, we assessed whether the resulting fingerprints contained clinical-group-associated structure across the IPMN-PDAC spectrum. This study therefore provides a comparative basis for selecting a nanoplatform configuration for subsequent prospective and longitudinal evaluation in the context of IPMN surveillance.

## 2. Materials and Methods

The study used a standardized SDS-PAGE-based PC fingerprinting pipeline designed to minimize operator-dependent variability and maintain compatibility with conventional laboratory instrumentation. The experimental and computational choices were based on previously reported SDS-PAGE/PC workflows and scripts [32,33,34].

### 2.1. Patients’ Enrollment and Inclusion Criteria

Blood samples were collected at the time of diagnosis during routine clinical work-up, before experimental processing. Sample collection, processing, and storage were performed in accordance with protocol 156.24 CET2 cbm, and written informed consent was obtained from all participants prior to enrollment. Demographic and clinical variables were recorded where available, including age, sex, comorbidities, smoking status, CA 19-9, radiologic features, histopathology, and stage.

Inclusion criteria were age ≥18 years, preserved hepatic and renal function, absence of active hematologic or infectious disease, and no previous malignancy other than pancreatic disease. PDAC diagnosis was confirmed by endoscopic ultrasound-guided fine-needle aspiration biopsy (EUS-FNAB) or by histopathology when surgical specimens were available. EUS-guided tissue acquisition represents an established approach for obtaining cytological or histological confirmation of solid pancreatic lesions [35,36]. Tumor staging followed the 8th edition of the AJCC TNM classification system, with pathological staging applied to resected cases and clinical staging used for unresectable disease. IPMN lesions were classified as main-duct, branch-duct, or mixed-type according to radiological, endoscopic, and pathological information, and the presence of worrisome features or high-risk stigmata was assessed according to international guideline criteria.

Plasma was obtained by centrifugation at 10 °C for 10 min at 1200× *g*, aliquoted, and stored at −80 °C until analysis. All samples were processed using the same incubation, washing, electrophoretic acquisition, and computational preprocessing pipeline within each nanoplatform.

### 2.2. Preparation of Graphene Oxide Nanoflakes

GO was purchased from Graphenea (San Sebastián, Spain). A 0.25 mg mL^−1^ aqueous dispersion was subjected to pulsed sonication for 2 min using a Vibra Cell VC505 sonicator (Sonics & Materials UK Ltd., Market Harborough, UK), yielding a homogeneous suspension of GO nanoflakes.

### 2.3. Preparation of the Liposome Formulation

The liposome formulation was prepared using DOTAP and DOPE (Avanti Polar Lipids, Alabaster, AL, USA) through the thin-film hydration method. The lipids were dissolved in chloroform, and the solvent was evaporated under vacuum to form a uniform lipid film on the inner walls of a glass vial. The dried film was then hydrated with ultrapure water to obtain a final lipid concentration of 1 mg mL^−1^, followed by brief vortexing to ensure complete resuspension. The resulting suspension was extruded 20 times through a 0.1 μm polycarbonate membrane using a Mini-Extruder (Avanti Polar Lipids, Alabaster, AL, USA). The final liposomal dispersions were stored at 4 °C until use.

### 2.4. Preparation of Gold Nanoparticles

Stabilized suspension of 100 nm AuNPs in citrate buffer (product ID: 742031) was purchased from Sigma Aldrich (St. Louis, MI, USA) and used without further treatment.

### 2.5. Preparation of NP-PC Complexes

Platform-specific NP-to-human plasma (HP) ratios were selected on the basis of previously established experimental conditions for the corresponding nanoplatforms. The present comparison therefore evaluated complete platform-specific analytical configurations rather than nanomaterial chemistry in isolation. One hundred microliters of GO solution was incubated with 0.5% (*v*/*v*) HP, whereas 50 μL of AuNPs and 50 μL of liposome suspension were each incubated with 50% (*v*/*v*) HP. All incubations were performed for 1 h at 37 °C.

Following incubation, samples were centrifuged at 18,620× *g* for 15 min at 4 °C, and the resulting pellets were washed three times with ultrapure water to remove unbound proteins. The purified NP-PC complexes were collected and stored at 4 °C for subsequent SDS-PAGE analysis.

### 2.6. Size and Zeta Potential Measurements

Naked NPs and representative NP-PC complexes from each disease group were diluted 100-fold in ultrapure water. Hydrodynamic size and zeta (ζ) potential were measured by DLS and micro-electrophoresis using a Zetasizer Nano ZS90 (Malvern Instruments Ltd., Malvern, UK). Measurements were performed at room temperature and reported as mean ± standard deviation of three independent experiments.

### 2.7. One-Dimensional SDS-PAGE Analysis

Following the washing steps, the pellets containing the NP-PC complexes were resuspended in Laemmli loading buffer under conditions optimized for each formulation. Specifically, 20 μL of 1× Laemmli buffer was used for GO and AuNPs, while 80 μL was employed for liposomes to ensure complete solubilization of the corona. Samples were then heated at 100 °C for 10 min and centrifuged at 18,620× *g* for 15 min at 4 °C. Subsequently, 10 μL of each supernatant was loaded onto stain-free 4–20% TGX precast polyacrylamide gels (Bio-Rad, Hercules, CA, USA) and electrophoresed at 150 V for approximately 90 min. Gel images were acquired using a ChemiDoc™ imaging system (Bio-Rad, Hercules, CA, USA). Gel images were processed with Image Lab(version 6.1, Bio-Rad Laboratories, Hercules, CA, USA) and custom MATLAB scripts (version R2023a, 9.14.0, MathWorks, Natick, MA, USA). One-dimensional lane intensity profiles were extracted after background correction and aligned to a common molecular weight (MW) axis. Each profile was normalized by dividing the lane intensity function by its total integral area over the entire MW domain, so that the normalized total area was equal to 1. Region-level features were then obtained by integrating normalized signal intensity over predefined MW windows. Because each normalized lane profile had a total area equal to 1, the resulting MW-region descriptors represent relative within-lane contributions rather than absolute protein abundance. Consequently, an increased normalized contribution in one MW region necessarily corresponds to a relative redistribution of signal across the remaining regions. All normalized MW-region differences were therefore interpreted as relative profile changes and not as absolute protein enrichment or depletion. No additional empirical batch-effect correction was applied after unit-area normalization and MW alignment.

### 2.8. Statistical Analysis and Classification Modeling

Quantitative descriptors corresponding to the integrated electrophoretic areas across the investigated MW ranges were used as input features for classification analysis. Linear discriminant analysis (LDA), implemented in MATLAB (Statistics and Machine Learning Toolbox, MathWorks, Natick, MA, USA), was used as an exploratory classification analysis. Given the limited cohort size and the unfavorable ratio between MW-region descriptors and training subjects in some folds, the resulting performance measures were interpreted as internal hypothesis-generating estimates rather than as precise or generalizable estimates of clinical diagnostic performance. To reduce dependence on a single arbitrary partition of this small and unbalanced cohort, model performance was assessed using repeated stratified k-fold cross-validation. Five-fold cross-validation was used for IPMN versus de novo PDAC, and three-fold cross-validation for comparisons involving IPMN-derived PDAC. For each repetition, samples were randomly partitioned into stratified folds; k − 1 folds were used for model training and the remaining fold was held out for testing. This process was continued until each fold had served once as the test set, and the complete cross-validation procedure was repeated 100 times using different random partitions. The repetitions reused the same 22 subjects and were not treated as independent observations or as an increase in the effective sample size. Within each cross-validation iteration, z-score standardization parameters were estimated exclusively from the training folds and subsequently applied to the corresponding held-out test fold. For each cross-validation iteration, classification performance was evaluated in terms of test accuracy, receiver operating characteristic (ROC) curve, and area under the ROC curve (AUC). Performance metrics were summarized as mean ± standard deviation across repeated cross-validation runs. The dispersion across repetitions represents cross-validation partition variability, namely sensitivity to the random allocation of subjects across folds, and should not be interpreted as a conventional population-level confidence interval or as uncertainty estimated from independent observations.

For descriptive region-level analyses, normalized MW-region areas were compared among the three clinical groups using one-way analysis of variance followed by Tukey’s multiple-comparison procedure. Tukey’s procedure controlled pairwise group comparisons within each individual MW region but did not provide global multiplicity control across the complete set of MW regions, nanoplatforms, and disease contrasts. Accordingly, all region-level *p*-values are reported as nominal and hypothesis-generating. The predefined MW regions were not selected on the basis of these *p*-values.

No multivariable adjustment for demographic or clinical characteristics was performed. Given the small class sizes, sparse covariate distributions, and complete or near-complete overlap between several clinical characteristics and diagnostic group, such an analysis would not permit stable separation of the individual covariate effects.

## 3. Results

The study cohort comprised 22 subjects encompassing the clinical groups under investigation. Demographic and clinical characteristics are summarized in Table 1. Each plasma specimen was independently interrogated using all three nanoplatform configurations: GO nanosheets, AuNPs, and cationic DOTAP-DOPE liposomes. This paired design enabled direct cross-platform comparison while maintaining the biological source and patient composition constant across nanoplatforms; it did not, however, remove demographic and clinical imbalances among diagnostic groups.

Physicochemical measurements obtained before and after plasma incubation are summarized in Table A1. Naked GO displayed a mean apparent hydrodynamic size of 775.3 ± 36.7 nm, a polydispersity index (PI) of 0.42 ± 0.07, and a ζ-potential of −46.8 ± 0.7 mV. Following plasma exposure, GO-containing complexes showed increased apparent sizes, ranging from 1237 to 3248 nm, together with PIs of 0.47–0.91 and attenuation of the negative surface charge to values between −43.2 and −21.9 mV. Naked AuNPs measured 126.1 ± 2.3 nm, with a PI of 0.15 ± 0.02 and a ζ-potential of −44.8 ± 2.5 mV; after plasma exposure, their size increased to 289–329 nm and the ζ-potential shifted to −29.1 to −24.0 mV. Naked DOTAP-DOPE liposomes measured 114.0 ± 0.1 nm and displayed a positive ζ-potential of +18.4 ± 2.1 mV. Plasma incubation increased their polydispersity and reversed the surface charge, yielding ζ-potentials between −22.0 and −14.4 mV. These changes were consistent with biomolecular adsorption and substantial alterations in the colloidal state of the nano–bio interfaces.

All three nanoplatforms produced recoverable wash-resistant PC fractions and structured one-dimensional SDS-PAGE profiles, but their electrophoretic architectures were non-equivalent (Figure 1).

GO profiles showed a prominent low-MW component with additional structured signal across intermediate- and high-MW regions. AuNP profiles were more concentrated in the intermediate-MW range and displayed several sharper secondary peaks. Liposome profiles showed a broad, multi-peak signal over the intermediate-MW range. These observations demonstrate material-dependent fractionation of the same plasma specimens but do not, by themselves, identify the underlying proteins or adsorption mechanisms. Unit-area normalization preserved the relative within-lane distribution of signal and enabled MW-window comparison across subjects, while removing information contained in absolute protein recovery.

Among the three complete analytical configurations, GO showed the broadest pattern of nominal clinical-group-associated variation in normalized MW-region contributions (Figure 2).

The 30–48 kDa region accounted for a nominally greater proportion of the total normalized lane signal in de novo PDAC than in both IPMN (*p* < 0.001) and IPMN-derived PDAC (*p* < 0.01). The 70–80 kDa region accounted for a nominally greater relative contribution in IPMN-derived PDAC than in both IPMN and de novo PDAC (*p* < 0.0001 for both comparisons). A nominal group-associated difference was also observed in the 150–250 kDa region, whose relative contribution was greater in IPMN-derived PDAC than in de novo PDAC (*p* < 0.05). The remaining GO-derived regions showed substantial overlap among groups.

The non-contiguous distribution of these nominal differences indicates that the observed group-associated pattern involved multiple relative MW regions. However, the underlying proteins and the clinical or biological factors responsible for these differences cannot be determined from the present analysis. GO-derived fingerprints showed different degrees of internally cross-validated separation across the three pairwise comparisons (Figure 3).

The numerically highest internal cross-validation AUC estimate was observed for IPMN versus de novo PDAC (AUC 0.955). The displayed subject-level confusion matrix classified all 6 IPMN samples and 9 of 11 de novo PDAC samples correctly. Posterior probabilities showed limited overlap, and the 30–48 kDa window accounted for most of the permutation importance, consistent with the corresponding group-level difference.

IPMN versus IPMN-derived PDAC was the most clinically proximate and the most difficult comparison (AUC 0.767). All 5 IPMN-derived PDAC samples were assigned to the malignant group, whereas only 3 of 6 non-invasive IPMN samples were assigned correctly in the displayed confusion matrix. The resulting asymmetry indicates that, at the illustrated decision threshold, sensitivity was favored at the cost of limited specificity. The 70–80 kDa window was the leading feature, followed by the 120–150 kDa interval, but posterior probabilities overlapped substantially.

GO fingerprints yielded an internal cross-validation AUC estimate of 0.927 for IPMN-derived versus de novo PDAC within this cohort. All 5 IPMN-derived PDAC samples and 8 of 11 de novo PDAC samples were assigned correctly in the displayed confusion matrix. The 70–80 and 150–250 kDa windows ranked highest in permutation importance.

Across the three GO comparisons, MW windows showing the largest group-associated differences were also among the leading held-out permutation features. This concordance provides exploratory internal agreement between the separately conducted region-level and classification analyses. However, it does not exclude covariance instability, overfitting, or confounding, and the feature rankings remain hypothesis-generating because the normalized MW-region areas are correlated and the sample size is small. The estimates obtained in comparisons involving IPMN-derived PDAC are particularly vulnerable to instability because some training folds contained only three or four subjects from one class.

AuNP-derived fingerprints showed fewer nominal group-associated differences than GO (Figure A1). The 60–72 kDa window was nominally lower in IPMN-derived than in de novo PDAC (*p* < 0.05), the 100–150 kDa window was nominally higher in IPMN-derived than in de novo PDAC (*p* < 0.01), and the 150–250 kDa window was nominally higher in IPMN-derived PDAC than in both non-invasive IPMN and de novo PDAC (*p* < 0.05 for each comparison). AuNP-based classification showed modest and inconsistent separation (Figure A2), with AUCs of 0.606 for IPMN versus de novo PDAC, 0.700 for IPMN versus IPMN-derived PDAC, and 0.636 for IPMN-derived versus de novo PDAC. Posterior-probability distributions remained broadly overlapping, and the leading permutation features were distributed across several intermediate- and high-MW windows.

DOTAP-DOPE liposome fingerprints showed the most restricted group-associated pattern (Figure A3). The only MW region showing a nominal group-associated difference at *p* < 0.05 was the 80–100 kDa window, which accounted for a nominally greater relative contribution in IPMN-derived than in de novo PDAC (*p* < 0.05). Liposome-based classification yielded AUCs of 0.621 for IPMN versus de novo PDAC, 0.633 for IPMN versus IPMN-derived PDAC, and 0.691 for IPMN-derived versus de novo PDAC (Figure A4). The 80–100 kDa window was the leading feature for IPMN-derived versus de novo PDAC, but subject-level posterior probabilities overlapped across all comparisons.

Across all three pairwise comparisons, GO yielded numerically higher internally cross-validated AUCs than AuNPs and liposomes: 0.955 versus 0.606 and 0.621 for IPMN versus de novo PDAC, 0.767 versus 0.700 and 0.633 for IPMN versus IPMN-derived PDAC, and 0.927 versus 0.636 and 0.691 for IPMN-derived versus de novo PDAC. Together with the broader pattern of nominal clinical-group-associated MW-region variation observed with GO, these paired exploratory findings supported prioritization of the GO-based configuration for subsequent replication and assay development. This prioritization applies to the specific experimental conditions tested and was not based on a formal multiplicity-controlled statistical comparison of platform performance.

Given the limited cohort size, these AUC values should be interpreted as internal estimates of within-cohort separation rather than as precise or generalizable estimates of clinical diagnostic performance.

## 4. Discussion

The principal finding of this exploratory study is that the same plasma specimens generated non-equivalent PC fingerprints when interrogated using three different nanoplatform configurations. Because each subject contributed plasma to all three analytical workflows, the biological source was held constant across the platform comparison. The observed differences therefore indicate that the analytical information recovered from plasma depends on the nano–bio interface and on the complete experimental configuration used to interrogate the sample. Among the tested configurations, GO showed the broadest pattern of nominal clinical-group-associated variation across predefined MW regions and yielded the numerically highest internal cross-validation estimates in all three pairwise comparisons. These findings support prioritization of the GO-based workflow for subsequent validation but do not establish its general superiority over other nanomaterials or validated clinical diagnostic performance.

This study extends previous work on NP-enabled blood testing beyond conventional PDAC-versus-control comparisons. Earlier studies demonstrated the feasibility of PC-based profiling using lipid NPs, AuNPs, and GO in pancreatic cancer and explored standardized electrophoretic workflows and integration with circulating biomarkers [25,26,27,28,29,30,31,32,37,38]. Here, the analytical question was different: whether distinct nanoplatform configurations could recover structured and non-equivalent information across the more biologically proximate and clinically heterogeneous IPMN-PDAC setting. This represents a more stringent context than cancer-versus-control classification because IPMN, IPMN-derived PDAC, and de novo PDAC may share inflammatory, stromal, metabolic, and systemic features. The clinical relevance of this setting lies in the longitudinal opportunity provided by IPMN surveillance. Patients with IPMN are identifiable before advanced pancreatic malignancy develops and may subsequently experience either progression of the monitored lesion to IPMN-derived carcinoma or development of a separate concomitant PDAC elsewhere in the pancreas. Within this context, a repeatable blood-derived molecular fingerprint could potentially be evaluated alongside established morphological and clinical criteria to determine whether it captures systemic biological changes not restricted to structural alterations of the known cyst. The present study does not demonstrate such a surveillance application; rather, it provides a rationale for testing this possibility prospectively.

The three pairwise comparisons should nevertheless be interpreted within their specific clinical contexts. IPMN versus de novo PDAC yielded the highest internal cross-validation AUC estimate (0.955) but also represented the largest clinical distance within the cohort. The numerical separation observed in this comparison may therefore reflect not only disease state but also differences in age, comorbidities, smoking exposure, tumor burden, biliary or inflammatory status, and CA 19-9. Eligibility criteria requiring preserved hepatic and renal function, absence of active hematologic or infectious disease, and no previous malignancy other than pancreatic disease limited some potential sources of systemic variability; however, they do not eliminate demographic, chronic clinical, or disease-related confounding. Accordingly, this result should not be interpreted as evidence that the contrast is intrinsically more clinically informative than comparisons involving more closely related neoplastic states.

The comparison between IPMN and IPMN-derived PDAC was more clinically proximate and yielded an internal AUC estimate of 0.767, with substantial overlap between groups. This is biologically plausible because epithelial and microenvironmental heterogeneity can emerge early in pancreatic precursor lesions, while progression from IPMN to invasive carcinoma involves complex molecular evolution rather than a uniform transition [39,40]. The observed overlap should therefore not be unexpected in an exploratory analysis of closely related disease states.

The comparison between IPMN-derived and de novo PDAC yielded an internal AUC estimate of 0.927, indicating that the exploratory model did not treat all malignant pancreatic conditions as analytically equivalent within this cohort. This observation is compatible with evidence that IPMN-associated carcinogenesis and conventional PDAC may differ in molecular and immune characteristics [8,40]. However, the present analysis cannot establish tumor lineage or clonal origin in individual patients. Differences in morphology, stage, ductal obstruction, systemic inflammation, and other clinical characteristics may also contribute to the observed separation. PC fingerprints should therefore be interpreted as composite group-associated systemic phenotypes rather than lineage-specific molecular signatures.

Across the GO analyses, the MW regions showing the strongest nominal group-associated differences were also among the leading features identified by held-out permutation analysis. The 30–48 kDa region contributed prominently to the IPMN versus de novo PDAC comparison, whereas the 70–80 kDa and higher-MW regions contributed more strongly to comparisons involving IPMN-derived PDAC. This concordance provides exploratory internal agreement between the region-level and classification analyses, but does not identify the molecular species responsible for the signal. SDS-PAGE MW regions are composite descriptors that may contain multiple proteins, proteolytic products, isoforms, modified species, and other molecular forms. Importantly, protein representation within the nanoparticle corona does not necessarily mirror bulk plasma abundance, because corona formation is governed by competitive adsorption, differential surface affinity, exchange kinetics, nanoparticle physicochemical properties, and the specific experimental conditions [19,20,21,22,23,24]. The resulting electrophoretic fingerprint should therefore not be interpreted as a direct readout of the abundance of a single dominant plasma protein. Direct proteomic characterization will therefore be required before these intervals can be assigned to specific proteins or biological pathways.

The broader group-associated variation observed with GO may plausibly reflect the characteristics of its two-dimensional oxidized surface. Oxygen-containing functional groups and residual aromatic domains allow electrostatic interactions, hydrogen bonding, hydrophobic association, dispersion forces, and π-π interactions to contribute simultaneously to biomolecular adsorption [41,42,43,44]. This heterogeneous interaction landscape may contribute to recruitment of a differently weighted plasma protein population. Nevertheless, the present experiment compared complete analytical configurations rather than nanomaterial chemistry in isolation. NP composition and geometry, plasma-to-particle ratio, colloidal behavior, washing conditions, recovery volume, solubilization, electrophoresis, and normalization all contributed to the final output. GO should therefore be regarded as the most informative configuration under the specific conditions tested here, rather than as a universally superior material for PC-based cancer profiling.

A methodological strength of this study is the paired within-specimen design, which reduces the possibility that cross-platform differences simply reflect differences in cohort composition. The SDS-PAGE-based readout was deliberately conceived as a low-complexity fingerprinting approach and may offer practical advantages for translational development because it requires relatively small plasma volumes, uses widely available laboratory instrumentation, and generates predefined MW-region descriptors that may facilitate standardization and interlaboratory transfer. These characteristics are consistent with the broader translational rationale of the REASSURED framework [45].

These advantages must, however, be considered alongside substantial limitations. The cohort was small and clinically imbalanced, particularly for IPMN-derived PDAC, and some training folds contained only three or four subjects from one class. Repeated cross-validation reduced dependence on a single train–test split but did not increase the effective sample size and cannot substitute for external validation. Reliable multivariable separation of demographic and clinical covariates was not feasible, and the resulting fingerprints should therefore be regarded as group-associated composite phenotypes rather than disease-specific signatures.

The analytical workflow also relied on unit-area normalization of electrophoretic profiles. This facilitated comparison of relative signal distributions but removed information on absolute protein recovery and imposed a constant-sum constraint on the MW-region descriptors. Group differences therefore represent changes in relative within-profile composition and should not be interpreted as absolute enrichment or depletion of proteins within individual MW regions. In addition, the cross-sectional design prevents inference regarding temporal progression. The present data cannot establish whether PC fingerprints precede radiological progression, predict future IPMN behavior, identify occult carcinoma, or provide incremental value over established surveillance strategies.

The next stage of development should therefore focus on independent replication in larger, prospectively annotated, multicenter cohorts representative of the clinical heterogeneity encountered during IPMN surveillance. Analytical procedures, quality-control criteria, and classification thresholds should be predefined, and samples should be prospectively annotated for relevant demographic and clinical variables. Longitudinal sampling during IPMN surveillance will be particularly important to determine whether GO-derived PC fingerprints remain stable in non-progressing disease and whether measurable changes occur in relation to clinical or radiological progression, the emergence of worrisome features or high-risk stigmata, IPMN-derived carcinoma, or concomitant PDAC. In parallel, mass-spectrometric characterization of the GO-derived corona across a broader electrophoretic range, with particular attention to regions identified as informative in the present analysis, will represent an essential component of subsequent biological validation, allowing the electrophoretic fingerprint to be linked to specific proteins and biological processes.

Ultimately, any future GO-based PC assay should be evaluated as a complementary biomarker layer rather than as a stand-alone diagnostic test. Its potential value will depend on whether molecular information obtained from plasma adds useful information to guideline-defined morphology, magnetic resonance imaging, endoscopic ultrasound, CA 19-9, cytology, histopathology, and other clinical variables. The present proof-of-concept study does not support changes to current IPMN surveillance or surgical decision-making but identifies a candidate analytical configuration and a testable framework for prospective longitudinal evaluation.

## 5. Conclusions

This paired proof-of-concept study demonstrated that different nanoplatform configurations generate non-equivalent plasma PC fingerprints across the investigated IPMN and PDAC groups. Under the tested conditions, GO showed the broadest nominal group-associated variation and the numerically highest internal discrimination estimates, supporting its prioritization for independent validation. Prospective longitudinal studies in larger, clinically overlapping IPMN populations, together with the appropriate assessment of clinical confounders and molecular characterization of the GO-derived corona, are required to determine whether GO-based PC profiling can provide complementary information for IPMN surveillance.

## Figures and Tables

**Figure 1:**
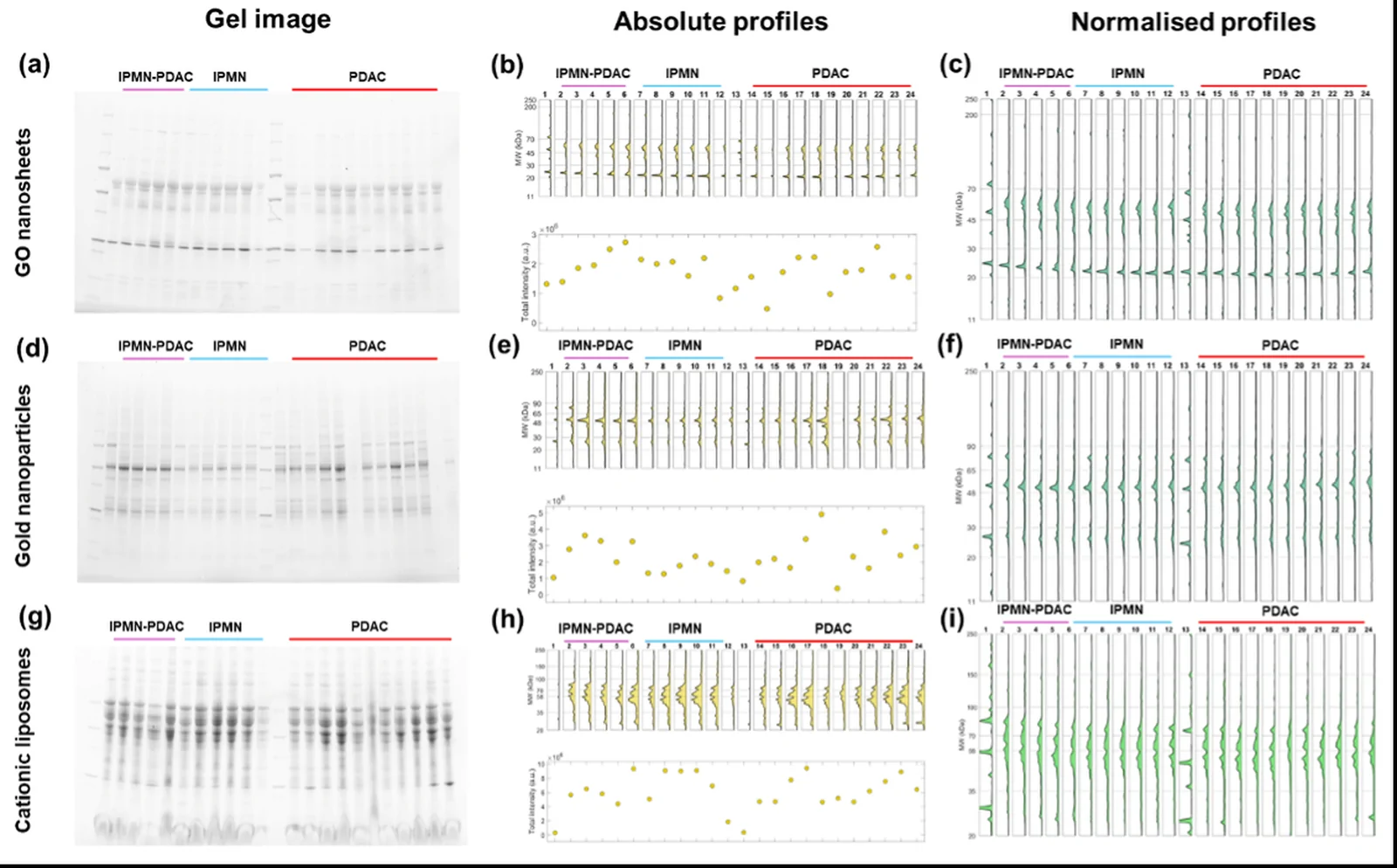
Nanomaterial-dependent architecture of plasma PC fingerprints. Representative SDS-PAGE gels and corresponding densitometric analyses after incubation of GO nanosheets, AuNPs, and cationic DOTAP-DOPE liposomes with plasma from patients with IPMN, IPMN-derived PDAC (IPMN-PDAC), and de novo PDAC (PDAC). Panels (**a**–**c**), (**d**–**f**), and (**g**–**i**) show GO, AuNP, and liposome results, respectively. Gel images are accompanied by absolute lane-intensity profiles and total-area-normalized profiles.

**Figure 2:**
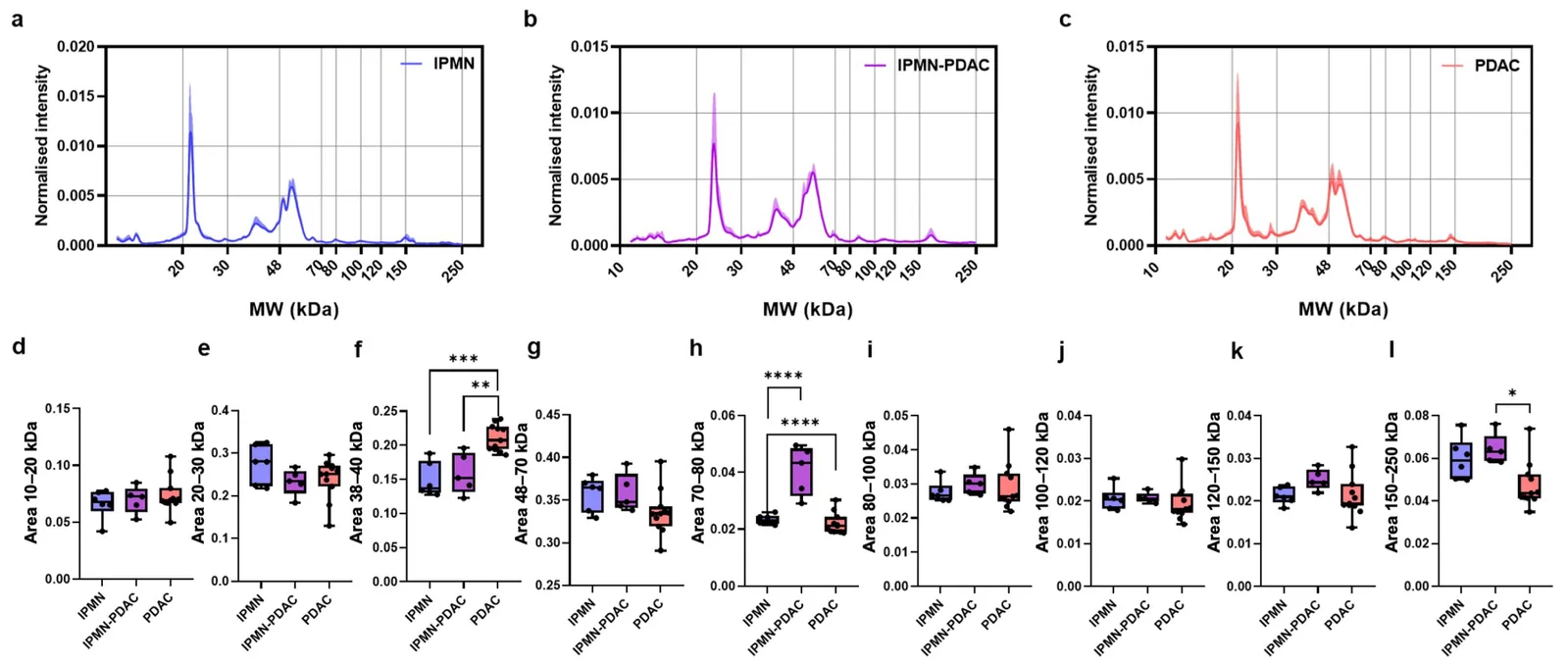
GO-derived electrophoretic profiles and molecular-weight-region analysis. (**a**) Normalized one-dimensional protein corona profile for IPMN; (**b**) normalized one-dimensional protein corona profile for IPMN-derived PDAC (IPMN-PDAC); (**c**) normalized one-dimensional protein corona profile for de novo PDAC; (**d**) integrated normalized area in the 10–20 kDa region; (**e**) integrated normalized area in the 20–30 kDa region; (**f**) integrated normalized area in the 30–48 kDa region; (**g**) integrated normalized area in the 48–70 kDa region; (**h**) integrated normalized area in the 70–80 kDa region; (**i**) integrated normalized area in the 80–100 kDa region; (**j**) integrated normalized area in the 100–120 kDa region; (**k**) integrated normalized area in the 120–150 kDa region; and (**l**) integrated normalized area in the 150–250 kDa region. Individual points represent patients. Group comparisons were performed using one-way analysis of variance followed by Tukey’s multiple-comparison test. * *p* < 0.05; ** *p* < 0.01; *** *p* < 0.001; **** *p* < 0.0001.

**Figure 3:**
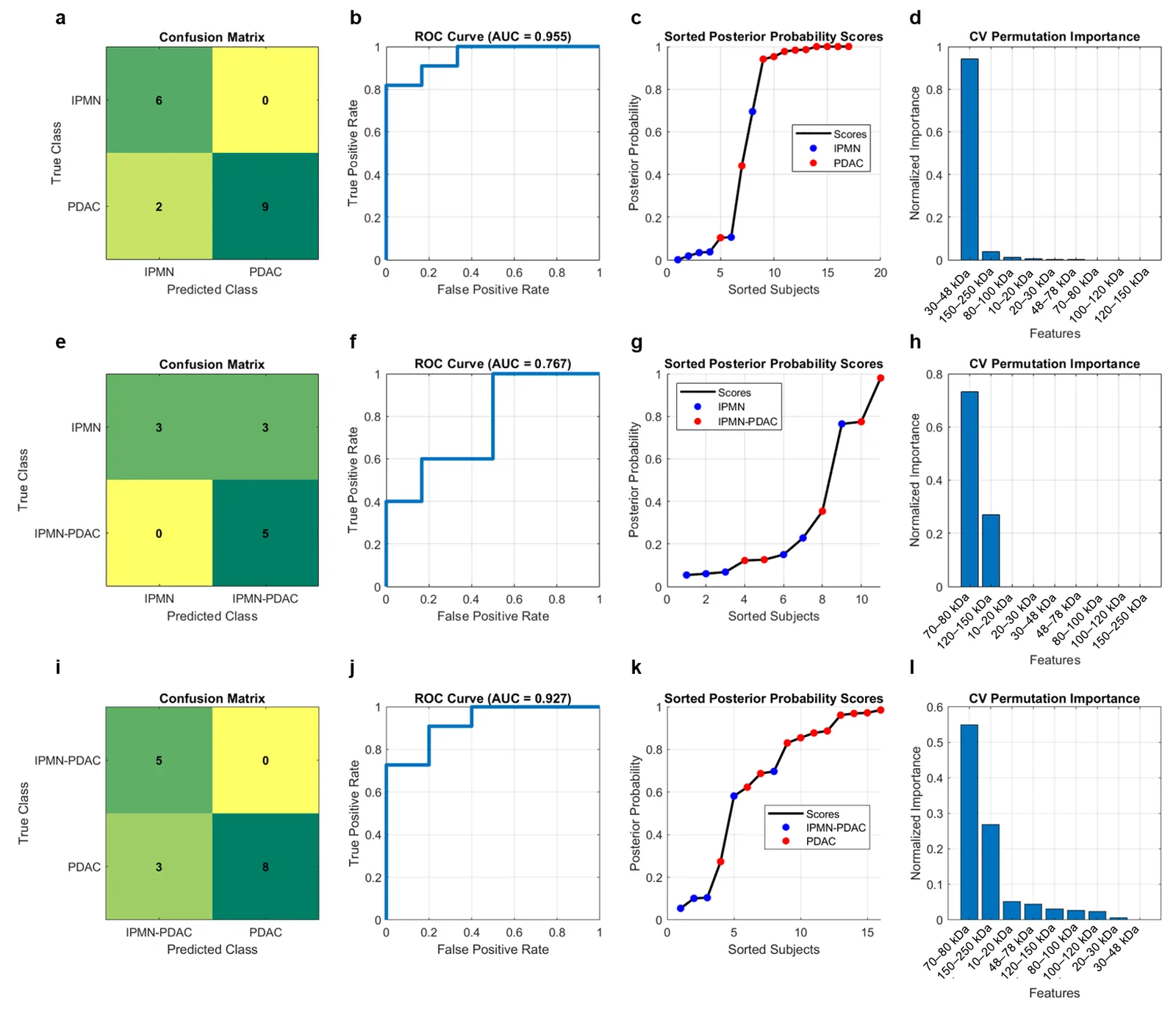
Cross-validated classification using GO-derived protein corona features. Pairwise linear discriminant analysis was performed for IPMN versus de novo PDAC (PDAC) (**a**–**d**), IPMN versus IPMN-derived PDAC (IPMN-PDAC) (**e**–**h**), and IPMN-derived PDAC versus de novo PDAC (**i**–**l**). For each comparison, the figure shows the confusion matrix, receiver operating characteristic curve with area under the curve, sorted posterior-probability scores, and cross-validated permutation feature importance calculated on held-out data. In the confusion matrices, cell colors represent the number of subjects in each true/predicted class combination, with yellow indicating lower counts and green indicating higher counts.

**Table 1:** Demographics and clinical characteristics of the clinical cohort.

	PDAC (*n* = 11)	IPMN (*n* = 6)	IPMN-Derived PDAC (*n* = 5)
**1. Demographics**			
Age, median (range), years	73 (60–83)	62 (49–85)	75 (64–85)
Sex, N (%)			
Male	7 (63.6)	3 (50.0)	3 (60.0)
Female	4 (36.4)	3 (50.0)	2 (40.0)
**2. Comorbidities, N (%)**			
Cardiac disease	7 (63.6)	0 (0)	3 (60.0)
Pulmonary disease	3 (27.3)	0 (0)	0 (0)
Diabetes mellitus	5 (45.5)	0 (0)	0 (0)
Hypertension	6 (54.5)	0 (0)	3 (60.0)
None	4 (36.4)	6 (100)	2 (40.0)
**3. Smoking status, N (%)**			
Never smoker	4 (36.4)	6 (100)	4 (80.0)
Former smoker	6 (54.5)	0 (0)	1 (20.0)
Current smoker	1 (9.1)	0 (0)	0 (0)
**4. Tumor-related characteristics ^1^**			
Tumor stage (AJCC 8th edition), N (%)			
Stage 0	0 (0)	NA	1 (20.0)
Stage I	5 (45.5)	NA	0 (0)
Stage II	1 (9.1)	NA	3 (60.0)
Stage III	4 (36.4)	NA	1 (20.0)
Stage IV	1 (9.1)	NA	0 (0)
**5. Biomarkers ^2^**			
CA 19-9 > 37 U/L, N (%)	10 (90.9)	NA	3 (60.0)
CA 19-9 ≤ 37 U/L, N (%)	1 (9.1)	NA	2 (40.0)
**6. IPMN-specific features ^3^**			
Worrisome features, N (%)	NA	1 (16.7)	0 (0)
High-risk stigmata present, N (%)	NA	0 (0)	5 (100)
**IPMN type ^3^, N (%)**			
Main-duct	NA	0 (0)	0 (0)
Branch-duct	NA	6 (100)	0 (0)
Mixed	NA	0 (0)	5 (100)

Data are presented as number (percentage) unless otherwise specified. NA indicates not applicable. ^1^ Tumor stage was assessed according to the AJCC 8th edition and evaluated only in patients with PDAC and IPMN-derived PDAC. ^2^ CA 19-9 values were dichotomized using a cut-off of 37 U/L. ^3^ IPMN-specific features and IPMN type were evaluated only in patients with IPMN and IPMN-derived PDAC.

**Figure A1:**
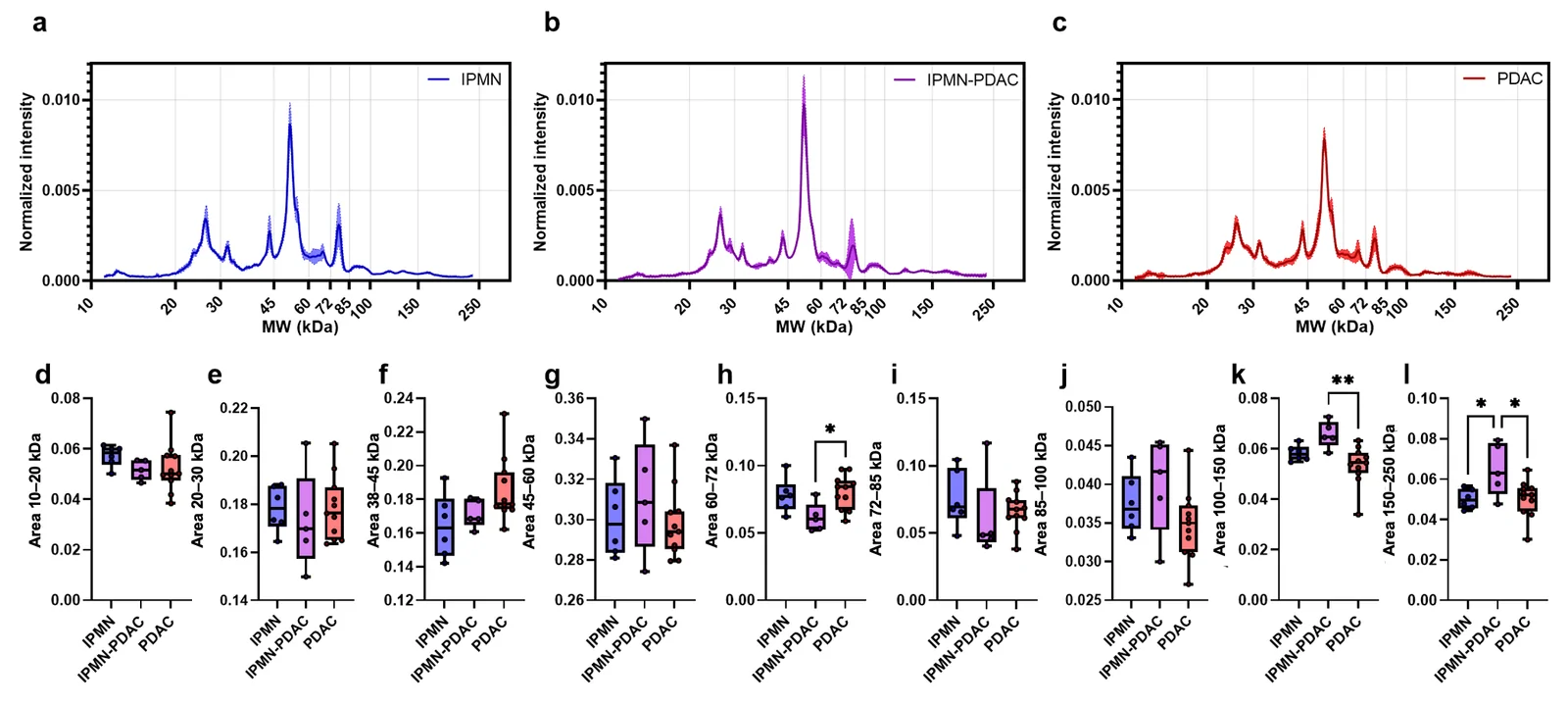
Electrophoretic outcomes of the protein corona formed on AuNPs after incubation with human plasma in the platform-selection cohort. (**a**) Normalized one-dimensional protein corona profile for IPMN; (**b**) normalized one-dimensional protein corona profile for IPMN-derived PDAC (IPMN-PDAC); (**c**) normalized one-dimensional protein corona profile for de novo PDAC; (**d**) integrated normalized area in the 10–20 kDa region; (**e**) integrated normalized area in the 20–30 kDa region; (**f**) integrated normalized area in the 30–45 kDa region; (**g**) integrated normalized area in the 45–60 kDa region; (**h**) integrated normalized area in the 60–72 kDa region; (**i**) integrated normalized area in the 72–85 kDa region; (**j**) integrated normalized area in the 85–100 kDa region; (**k**) integrated normalized area in the 100–150 kDa region; and (**l**) integrated normalized area in the 150–250 kDa region. Individual points represent patients. Group comparisons were performed using one-way analysis of variance followed by Tukey’s multiple-comparison test. * *p* < 0.05; ** *p* < 0.01.

**Figure A2:**
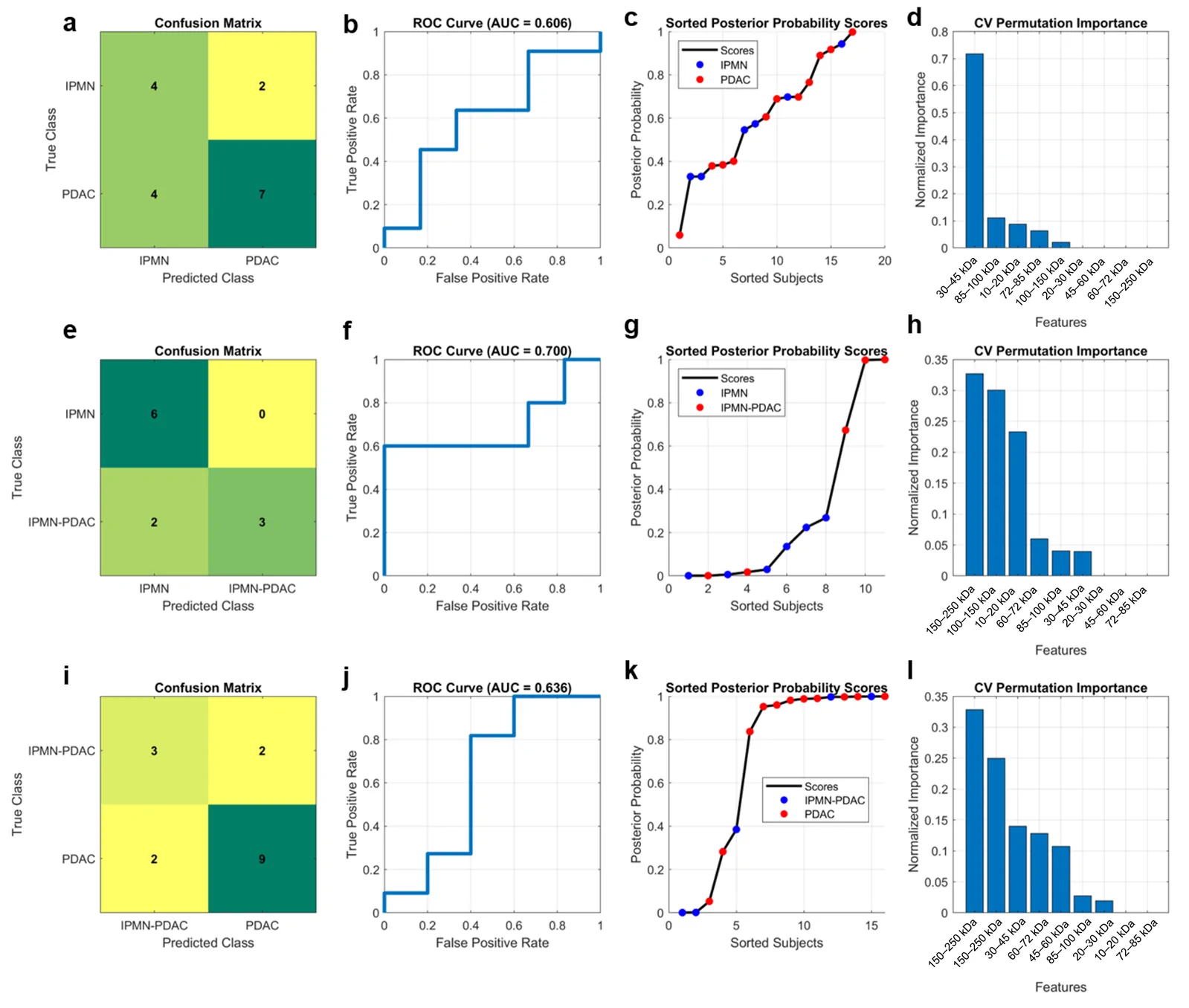
AuNP-based classification performance and feature-importance analysis in the platform-selection cohort. Pairwise linear discriminant analysis was performed for IPMN versus de novo PDAC (PDAC) (**a**–**d**), IPMN versus IPMN-derived PDAC (IPMN-PDAC) (**e**–**h**), and IPMN-derived PDAC versus de novo PDAC (**i**–**l**). For each comparison, the figure shows the confusion matrix, receiver operating characteristic curve with area under the curve, sorted posterior-probability scores, and cross-validated permutation feature importance calculated on held-out data. In the confusion matrices, cell colors represent the number of subjects in each true/predicted class combination, with yellow indicating lower counts and green indicating higher counts.

**Figure A3:**
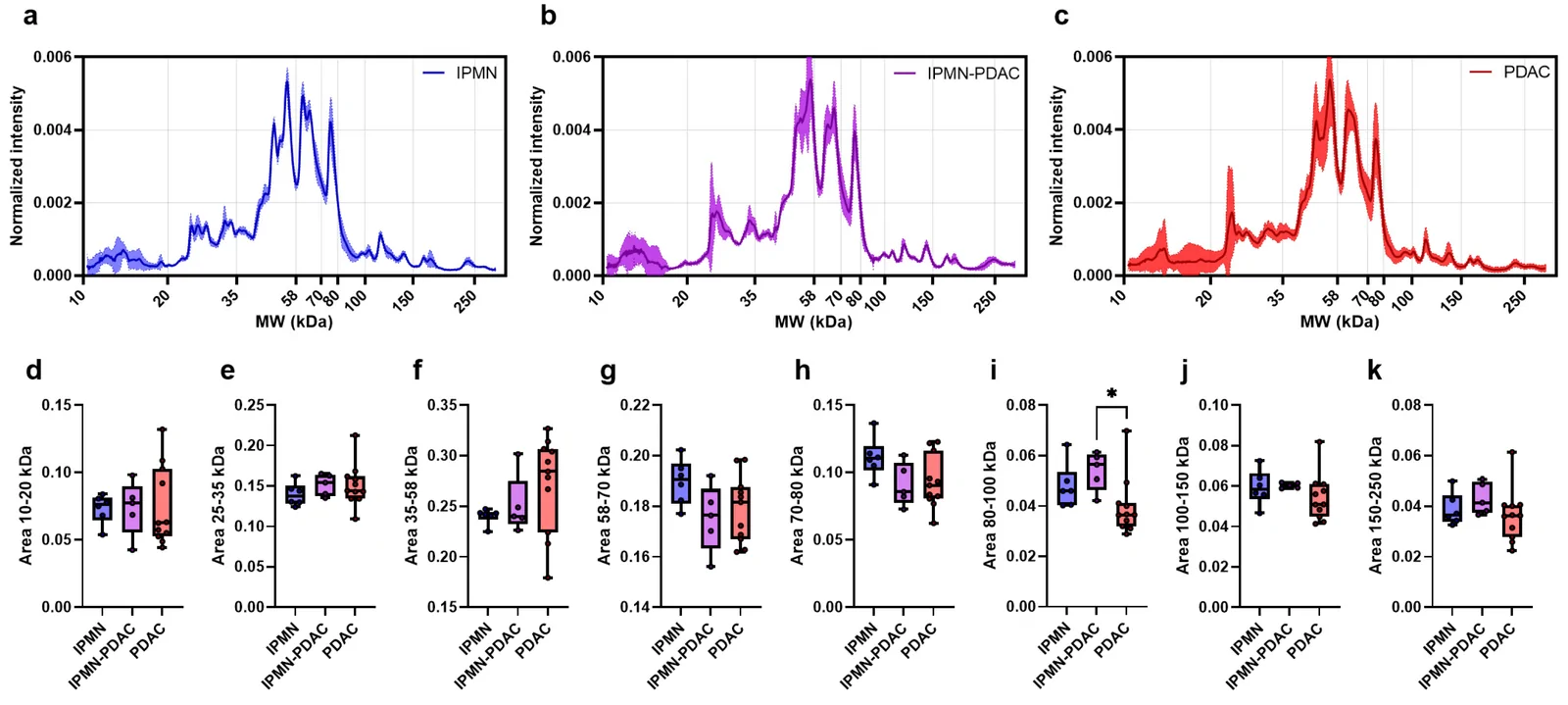
Electrophoretic outcomes of the protein corona formed on DOTAP-DOPE liposomes after incubation with human plasma in the platform-selection cohort. (**a**) Normalized one-dimensional protein corona profile for IPMN; (**b**) normalized one-dimensional protein corona profile for IPMN-derived PDAC (IPMN-PDAC); (**c**) normalized one-dimensional protein corona profile for de novo PDAC; (**d**) integrated normalized area in the 10–20 kDa region; (**e**) integrated normalized area in the 25–35 kDa region; (**f**) integrated normalized area in the 35–58 kDa region; (**g**) integrated normalized area in the 58–70 kDa region; (**h**) integrated normalized area in the 70–80 kDa region; (**i**) integrated normalized area in the 80–100 kDa region; (**j**) integrated normalized area in the 100–150 kDa region; and (**k**) integrated normalized area in the 150–250 kDa region. Individual points represent patients. Group comparisons were performed using one-way analysis of variance followed by Tukey’s multiple-comparison test.

**Figure A4:**
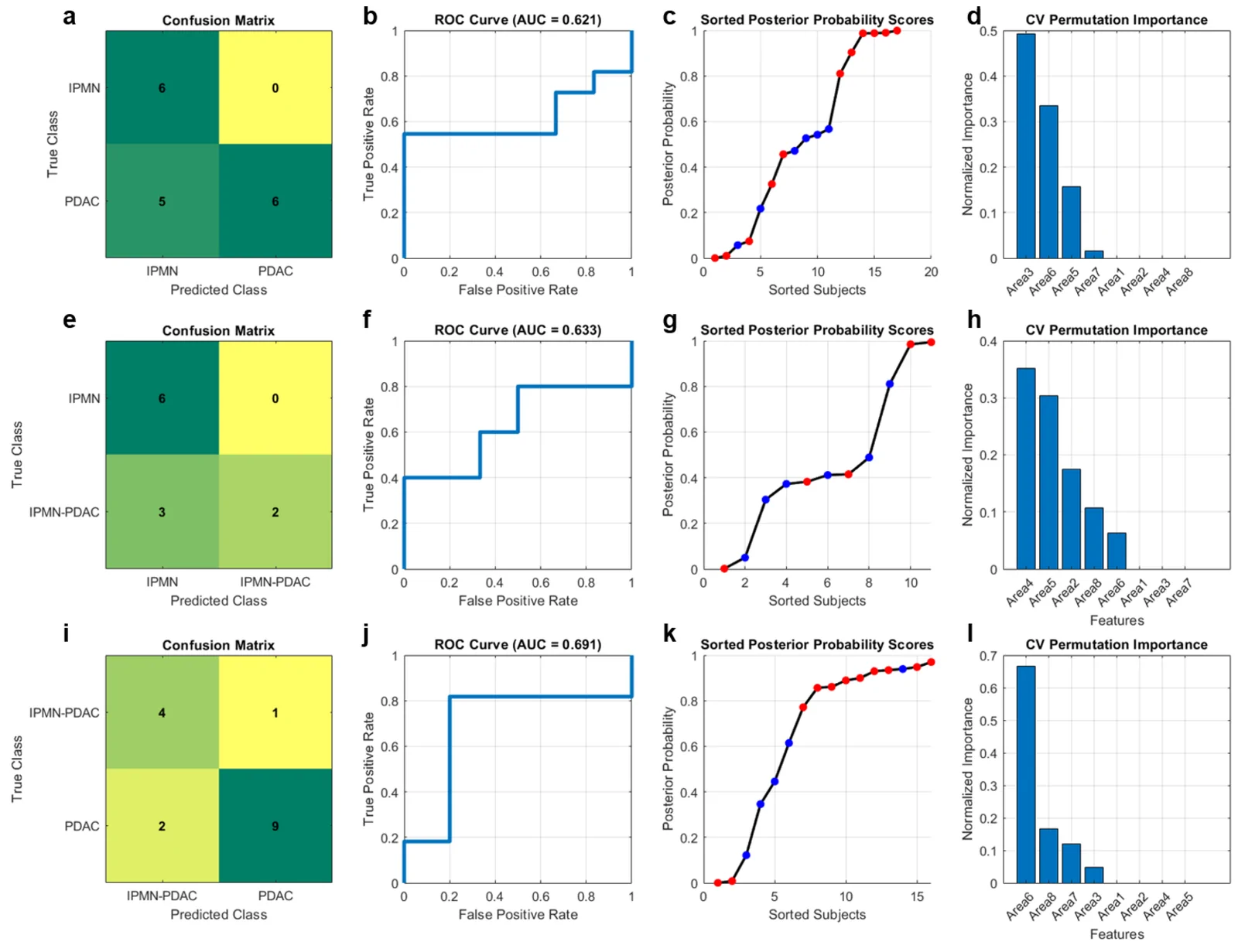
DOTAP-DOPE liposome-based classification performance and feature-importance analysis in the platform-selection cohort. Pairwise linear discriminant analysis was performed for IPMN versus de novo PDAC (PDAC) (**a**–**d**), IPMN versus IPMN-derived PDAC (IPMN-PDAC) (**e**–**h**), and IPMN-derived PDAC versus de novo PDAC (**i**–**l**). For each comparison, the figure shows the confusion matrix, receiver operating characteristic curve with area under the curve, sorted posterior-probability scores, and cross-validated permutation feature importance calculated on held-out data. In the confusion matrices, cell colors represent the number of subjects in each true/predicted class combination, with yellow indicating lower counts and green indicating higher counts.

## Data Availability

The original contributions presented in the study are included in the article. Further inquiries can be directed to the corresponding author.

## Abbreviations

The following abbreviations are used in this manuscript:

AJCC	American Joint Committee on Cancer
ANOVA	Analysis of Variance
AUC	Area Under the Receiver Operating Characteristic Curve
AuNPs	Gold Nanoparticles
CA 19-9	Carbohydrate Antigen 19-9
DLS	Dynamic Light Scattering
DOPE	1,2-Dioleoyl-sn-glycero-3-phosphoethanolamine
DOTAP	1,2-Dioleoyl-3-trimethylammonium-propane
EUS-FNAB	Endoscopic Ultrasound-Guided Fine-Needle Aspiration Biopsy
GO	Graphene Oxide
HP	Human Plasma
IPMN	Intraductal Papillary Mucinous Neoplasm
IPMN-PDAC	IPMN-Derived Pancreatic Ductal Adenocarcinoma
LDA	Linear Discriminant Analysis
MW	Molecular Weight
NP	Nanoparticle
NP-PC	Nanoparticle–Protein Corona
PC	Protein Corona
PDAC	Pancreatic Ductal Adenocarcinoma
PI	Polydispersity Index
ROC	Receiver Operating Characteristic
SDS-PAGE	Sodium Dodecyl Sulfate–Polyacrylamide Gel Electrophoresis
TNM	Tumor–Node–Metastasis

## Appendix A. Additional Nanoplatform Characterization and Comparative Analyses

Appendix A provides additional physicochemical characterization and platform-specific analyses supporting the comparative evaluation of graphene oxide (GO), gold nanoparticles (AuNPs), and DOTAP–DOPE liposomes described in the main text.

### Appendix A.1. Physicochemical Characterization of Nanoparticle–Protein Corona Complexes

**Table A1:** Dynamic light scattering (DLS) characterization of graphene oxide (GO) nanosheets, gold nanoparticles (AuNPs), and cationic DOTAP–DOPE liposomes before and after incubation with representative plasma samples. Hydrodynamic size, polydispersity index (PI), and zeta potential are reported. Plasma samples from the same representative subjects were analyzed for comparison.

Sample	Size (nm)	Polydispersity Index (PI)	Zeta Potential (mV)
IPMN-PDAC	239.4 ± 12.4	0.7 ± 0.2	−23.4 ± 1.4
IPMN	271.1 ± 35.2	0.78 ± 0.3	−26.4 ± 2.3
PDAC	255.7 ± 20.3	0.84 ± 0.3	−28.3 ± 3.2
GO nanosheets	775.3 ± 36.7	0.42 ± 0.07	−46.8 ± 0.68
GO 0.5% + IPMN-PDAC	3248 ± 509	0.91 ± 0.07	−23.13 ± 2.77
GO 0.5% + IPMN	3213 ± 281	0.80 ± 0.28	−21.86 ± 0.61
GO 0.5% + PDAC	1237 ± 25.46	0.47 ± 0.07	−43.16 ± 0.35
Au nanoparticle	126.07 ± 2.25	0.15 ± 0.02	−44.8 ± 2.5
Au 50% + IPMN-PDAC	291.4 ± 60.67	0.61 ± 0.14	−28.62 ± 0.42
Au 50% + IPMN	328.55 ± 22.27	0.44 ± 0.05	−29.14 ± 0.33
Au 50% + PDAC	289.15 ± 3.75	0.59 ± 0.05	−23.97 ± 0.98
DOTAP-DOPE liposome	114 ± 0.13	0.06 ± 0.012	18.4 ± 2.07
DOTAP-DOPE 50% + IPMN-PDAC	104.75 ± 26.09	0.51 ± 0.04	−21.96 ± 1.09
DOTAP-DOPE 50% + IPMN	385.20 ± 22.49	0.54 ± 0.05	−18.75 ± 0.49
DOTAP-DOPE 50% + PDAC	443.55 ± 96.38	0.49 ± 0.03	−14.41 ± 0.76

## References

[1] Stoop T.F., Javed A.A., Oba A., Koerkamp B.G., Seufferlein T., Wilmink J.W., Besselink M.G. (2025). Pancreatic Cancer. Lancet.

[2] Halbrook C.J., Lyssiotis C.A., Pasca di Magliano M., Maitra A. (2023). Pancreatic Cancer: Advances and Challenges. Cell.

[3] Nakaoka K., Ohno E., Kawabe N., Kuzuya T., Funasaka K., Nakagawa Y., Nagasaka M., Ishikawa T., Watanabe A., Tochio T. (2023). Current Status of the Diagnosis of Early-Stage Pancreatic Ductal Adenocarcinoma. Diagnostics.

[4] Ohtsuka T., Fernandez-del Castillo C., Furukawa T., Hijioka S., Jang J.-Y., Lennon A.M., Miyasaka Y., Ohno E., Salvia R., Wolfgang C.L. (2024). International Evidence-Based Kyoto Guidelines for the Management of Intraductal Papillary Mucinous Neoplasm of the Pancreas. Pancreatology.

[5] (2018). The European Study Group on Cystic Tumours of the Pancreas. European Evidence-Based Guidelines on Pancreatic Cystic Neoplasms. Gut.

[6] Oyama H., Tada M., Takagi K., Tateishi K., Hamada T., Nakai Y., Hakuta R., Ijichi H., Ishigaki K., Kanai S. (2020). Long-Term Risk of Malignancy in Branch-Duct Intraductal Papillary Mucinous Neoplasms. Gastroenterology.

[7] Oyama H., Hamada T., Nakai Y., Tanaka M., Takagi K., Fukuda R., Hakuta R., Ishigaki K., Kanai S., Kawaguchi Y. (2025). Intraductal Papillary Mucinous Neoplasm Surveillance Leads to Early Diagnosis and Better Outcomes of Concomitant Cancer. Ann. Surg..

[8] Park M.A., Gumpper-Fedus K., Krishna S.G., Genilo-Delgado M.C., Brantley S., Hart P.A., Dillhoff M.E., Gomez M.F., Basinski T.L., Mok S.R. (2024). Molecular Pathway and Immune Profile Analysis of IPMN-Derived Versus PanIN-Derived Pancreatic Ductal Adenocarcinomas. Int. J. Mol. Sci..

[9] Fogliati A., Zironda A., Fiorentini G., Adjei S., Amro A., Starlinger P.P., Grotz T.E., Warner S.G., Smoot R.L., Thiels C.A. (2024). Outcomes of Neoadjuvant Chemotherapy for Invasive Intraductal Papillary Mucinous Neoplasm Compared with de Novo Pancreatic Adenocarcinoma. Ann. Surg. Oncol..

[10] Coppola A., La Vaccara V., Fiore M., Farolfi T., Ramella S., Angeletti S., Coppola R., Caputo D. (2021). CA19.9 Serum Level Predicts Lymph-Nodes Status in Resectable Pancreatic Ductal Adenocarcinoma: A Retrospective Single-Center Analysis. Front. Oncol..

[11] Lee T., Teng T.Z.J., Shelat V.G. (2020). Carbohydrate Antigen 19-9—Tumor Marker: Past, Present, and Future. World J. Gastrointest. Surg..

[12] Bettegowda C., Sausen M., Leary R.J., Kinde I., Wang Y., Agrawal N., Bartlett B.R., Wang H., Luber B., Alani R.M. (2014). Detection of Circulating Tumor DNA in Early- and Late-Stage Human Malignancies. Sci. Transl. Med..

[13] Cohen J.D., Javed A.A., Thoburn C., Wong F., Tie J., Gibbs P., Schmidt C.M., Yip-Schneider M.T., Allen P.J., Schattner M. (2017). Combined Circulating Tumor DNA and Protein Biomarker-Based Liquid Biopsy for the Earlier Detection of Pancreatic Cancers. Proc. Natl. Acad. Sci. USA.

[14] Cohen J.D., Li L., Wang Y., Thoburn C., Afsari B., Danilova L., Douville C., Javed A.A., Wong F., Mattox A. (2018). Detection and Localization of Surgically Resectable Cancers with a Multi-Analyte Blood Test. Science (1979).

[15] Brand R.E., Persson J., Bratlie S.O., Chung D.C., Katona B.W., Carrato A., Castillo M., Earl J., Kokkola A., Lucas A.L. (2022). Detection of Early-Stage Pancreatic Ductal Adenocarcinoma From Blood Samples: Results of a Multiplex Biomarker Signature Validation Study. Clin. Transl. Gastroenterol..

[16] Peterka O., Jirásko R., Dolečková Z., Dosoudilová M., Bártl J., Idkowiak J., Slavíček O., Pešková K., Vošmik M., Mohelníková-Duchoňová B. (2026). Pilot Study of Screening Method for Pancreatic Cancer Using Lipidomic Profiling of Plasma or Serum. Commun. Med..

[17] Mahajan U.M., Oehrle B., Goni E., Strobel O., Kaiser J., Grützmann R., Werner J., Friess H., Gress T.M., Seufferlein T.W. (2025). Validation of Two Plasma Multimetabolite Signatures for Patients at Risk of or with Suspected Pancreatic Ductal Adenocarcinoma (METAPAC): A Prospective, Multicentre, Investigator-Masked, Enrichment Design, Phase 4 Diagnostic Study. Lancet Gastroenterol. Hepatol..

[18] Devasahayam Arokia Balaya R., Sen P., Grant C.W., Zenka R., Sappani M., Lakshmanan J., Athreya A.P., Kandasamy R.K., Pandey A., Byeon S.K. (2025). An Integrative Multi-Omics Analysis Reveals a Multi-Analyte Signature of Pancreatic Ductal Adenocarcinoma in Serum. J. Gastroenterol..

[19] Monopoli M.P., Åberg C., Salvati A., Dawson K.A. (2012). Biomolecular Coronas Provide the Biological Identity of Nanosized Materials. Nat. Nanotechnol..

[20] Tenzer S., Docter D., Kuharev J., Musyanovych A., Fetz V., Hecht R., Schlenk F., Fischer D., Kiouptsi K., Reinhardt C. (2013). Rapid Formation of Plasma Protein Corona Critically Affects Nanoparticle Pathophysiology. Nat. Nanotechnol..

[21] Cedervall T., Lynch I., Lindman S., Berggård T., Thulin E., Nilsson H., Dawson K.A., Linse S. (2007). Understanding the Nanoparticle–Protein Corona Using Methods to Quantify Exchange Rates and Affinities of Proteins for Nanoparticles. Proc. Natl. Acad. Sci. USA.

[22] Lundqvist M., Stigler J., Elia G., Lynch I., Cedervall T., Dawson K.A. (2008). Nanoparticle Size and Surface Properties Determine the Protein Corona with Possible Implications for Biological Impacts. Proc. Natl. Acad. Sci. USA.

[23] Blume J.E., Manning W.C., Troiano G., Hornburg D., Figa M., Hesterberg L., Platt T.L., Zhao X., Cuaresma R.A., Everley P.A. (2020). Rapid, Deep and Precise Profiling of the Plasma Proteome with Multi-Nanoparticle Protein Corona. Nat. Commun..

[24] González-García L.E., MacGregor M.N., Visalakshan R.M., Lazarian A., Cavallaro A.A., Morsbach S., Mierczynska-Vasilev A., Mailänder V., Landfester K., Vasilev K. (2022). Nanoparticles Surface Chemistry Influence on Protein Corona Composition and Inflammatory Responses. Nanomaterials.

[25] Caputo D., Papi M., Coppola R., Palchetti S., Digiacomo L., Caracciolo G., Pozzi D. (2017). A Protein Corona-Enabled Blood Test for Early Cancer Detection. Nanoscale.

[26] Palchetti S., Caputo D., Digiacomo L., Capriotti A.L., Coppola R., Pozzi D., Caracciolo G. (2019). Protein Corona Fingerprints of Liposomes: New Opportunities for Targeted Drug Delivery and Early Detection in Pancreatic Cancer. Pharmaceutics.

[27] Digiacomo L., Caputo D., Coppola R., Cascone C., Giulimondi F., Palchetti S., Pozzi D., Caracciolo G. (2021). Efficient Pancreatic Cancer Detection through Personalized Protein Corona of Gold Nanoparticles. Biointerphases.

[28] Di Santo R., Digiacomo L., Quagliarini E., Capriotti A.L., Laganà A., Zenezini Chiozzi R., Caputo D., Cascone C., Coppola R., Pozzi D. (2020). Personalized Graphene Oxide-Protein Corona in the Human Plasma of Pancreatic Cancer Patients. Front. Bioeng. Biotechnol..

[29] Caputo D., Coppola A., Quagliarini E., Di Santo R., Capriotti A.L., Cammarata R., Laganà A., Papi M., Digiacomo L., Coppola R. (2022). Multiplexed Detection of Pancreatic Cancer by Combining a Nanoparticle-Enabled Blood Test and Plasma Levels of Acute-Phase Proteins. Cancers.

[30] Digiacomo L., Quagliarini E., Pozzi D., Coppola R., Caracciolo G., Caputo D. (2023). Stratifying Risk for Pancreatic Cancer by Multiplexed Blood Test. Cancers.

[31] Digiacomo L., Caputo D., Cammarata R., La Vaccara V., Coppola R., Quagliarini E., Iacobini M., Renzi S., Giulimondi F., Pozzi D. (2025). Nanoparticle-Protein Corona Enhances Accuracy of Ca-19.9-Based Pancreatic Cancer Classification. Nanoscale.

[32] Quagliarini E., Digiacomo L., Giulimondi F., Renzi S., Iacobini M., Caracciolo G., Pozzi D. (2025). Standardized SDS-PAGE Workflow for Personalized Protein Corona Profiling in Early Cancer Detection. J. Vis. Exp..

[33] Renzi S., Digiacomo L., Pozzi D., Quagliarini E., Vulpis E., Giuli M.V., Mancusi A., Natiello B., Pignataro M.G., Canettieri G. (2024). Structuring Lipid Nanoparticles, DNA, and Protein Corona into Stealth Bionanoarchitectures for in Vivo Gene Delivery. Nat. Commun..

[34] Digiacomo L., Palchetti S., Giulimondi F., Pozzi D., Zenezini Chiozzi R., Capriotti A.L., Laganà A., Caracciolo G. (2019). The Biomolecular Corona of Gold Nanoparticles in a Controlled Microfluidic Environment. Lab Chip.

[35] Facciorusso A., Stasi E., Di Maso M., Serviddio G., Ali Hussein M.S., Muscatiello N. (2017). Endoscopic Ultrasound-guided Fine Needle Aspiration of Pancreatic Lesions with 22 versus 25 Gauge Needles: A Meta-analysis. United Eur. Gastroenterol. J..

[36] Mangiavillano B., Crinò S.F., Facciorusso A., Di Matteo F., Barbera C., Larghi A., Rizzatti G., Carrara S., Spadaccini M., Auriemma F. (2023). Endoscopic Ultrasound-Guided Fine-Needle Biopsy with or without Macroscopic on-Site Evaluation: A Randomized Controlled Noninferiority Trial. Endoscopy.

[37] Caputo D., Cartillone M., Cascone C., Pozzi D., Digiacomo L., Palchetti S., Caracciolo G., Coppola R. (2018). Improving the Accuracy of Pancreatic Cancer Clinical Staging by Exploitation of Nanoparticle-Blood Interactions: A Pilot Study. Pancreatology.

[38] Papi M., Palmieri V., Digiacomo L., Giulimondi F., Palchetti S., Ciasca G., Perini G., Caputo D., Cartillone M.C., Cascone C. (2019). Converting the Personalized Biomolecular Corona of Graphene Oxide Nanoflakes into a High-Throughput Diagnostic Test for Early Cancer Detection. Nanoscale.

[39] Bernard V., Semaan A., Huang J., San Lucas F.A., Mulu F.C., Stephens B.M., Guerrero P.A., Huang Y., Zhao J., Kamyabi N. (2019). Single-Cell Transcriptomics of Pancreatic Cancer Precursors Demonstrates Epithelial and Microenvironmental Heterogeneity as an Early Event in Neoplastic Progression. Clin. Cancer Res..

[40] Omori Y., Ono Y., Tanino M., Karasaki H., Yamaguchi H., Furukawa T., Enomoto K., Ueda J., Sumi A., Katayama J. (2019). Pathways of Progression From Intraductal Papillary Mucinous Neoplasm to Pancreatic Ductal Adenocarcinoma Based on Molecular Features. Gastroenterology.

[41] Liu X., Yan C., Chen K.L. (2019). Adsorption of Human Serum Albumin on Graphene Oxide: Implications for Protein Corona Formation and Conformation. Environ. Sci. Technol..

[42] Baruah A., Newar R., Das S., Kalita N., Nath M., Ghosh P., Chinnam S., Sarma H., Narayan M. (2024). Biomedical Applications of Graphene-Based Nanomaterials: Recent Progress, Challenges, and Prospects in Highly Sensitive Biosensors. Discov. Nano.

[43] Chong Y., Ge C., Yang Z., Garate J.A., Gu Z., Weber J.K., Liu J., Zhou R. (2015). Reduced Cytotoxicity of Graphene Nanosheets Mediated by Blood-Protein Coating. ACS Nano.

[44] Gu Z., Yang Z., Wang L., Zhou H., Jimenez-Cruz C.A., Zhou R. (2015). The Role of Basic Residues in the Adsorption of Blood Proteins onto the Graphene Surface. Sci. Rep..

[45] Land K.J., Boeras D.I., Chen X.-S., Ramsay A.R., Peeling R.W. (2018). REASSURED Diagnostics to Inform Disease Control Strategies, Strengthen Health Systems and Improve Patient Outcomes. Nat. Microbiol..

